# Higher Apnea-Hypopnea Index (AHI) and Oxygen Desaturation Index (ODI) Were Independently Associated with Increased Risks of Hypertension in Patients with T2DM: A Cross-Sectional Study

**DOI:** 10.1155/2021/8887944

**Published:** 2021-01-21

**Authors:** Weijuan Su, Guobing Chen, Danyan Ma, Jinyang Zeng, Fangfang Yan, Xiaoyan Lin, Ziqing Xu, Shuyu Yang, Zhibin Li, Changqin Liu

**Affiliations:** ^1^Department of Endocrinology and Diabetes, The First Affiliated Hospital of Xiamen University, Xiamen, China; ^2^Xiamen Clinical Medical Center for Endocrine and Metabolic Diseases, Fujian Province Key Laboratory of Diabetes Translational Medicine, Xiamen, China; ^3^Department of Pediatrics, The First Affiliated Hospital of Xiamen University, Xiamen, China; ^4^School of Medicine, Xiamen University, Xiamen, China; ^5^Xiamen Diabetes Institute, The First Affiliated Hospital, Xiamen University, Xiamen, China; ^6^Epidemiology Research Unit, Center of Translational Medical Research, The First Affiliated Hospital of Xiamen University, Xiamen, China; ^7^The School of Clinical Medicine, Fujian Medical University, Fuzhou, China

## Abstract

**Objective:**

The current study aimed to explore the relationship between OSAS and hypertension and whether polysomnography (PSG) indices were independently associated with hypertension in patients with type 2 diabetes (T2DM).

**Methods:**

This study recruited 316 T2DM patients. Multivariable logistic regression analyses were performed to determine the independent association of apnea-hypopnea index (AHI) and oxygen desaturation index (ODI) with hypertension with adjustment for potential confounders.

**Results:**

Among 316 patients, 130 (41.1%) and 204 (64.6%) had hypertension and OSAS, respectively. T2DM patients with hypertension showed significantly increased levels of AHI ((median (interquartile range)): 17.2 (5.7–34.9) vs. 5.7 (2.1–17.3) events/hour, *p* < 0.001), nonrapid eye movement AHI (NREM-AHI) (17.6 (5.5–36.5) vs. 5.2 (2.2–16.6) events/hour, *p* < 0.001), ODI (48.4 (21.9–78.0) vs. 22.6 (10.8–48.1) events/hour, *p* < 0.001), and severities of OSAS and decreased levels of lowest SaO_2_ ((mean ± standard deviation): 74.0 ± 10.4 vs. 77.3 ± 9.8, *p* = 0.004). Multivariable logistic regression analysis showed that higher levels of AHI, NREM-AHI, and ODI were significantly associated with increased risks of hypertension, and the adjusted odds ratios (ORs) with 95% CI were 1.026 (1.008–1.044, *p* = 0.004), 1.026 (1.009–1.044, *p* = 0.003), and 1.005 (1.001–1.010, *p* = 0.040), respectively. Compared with non-OSAS, severe OSAS was significantly associated with the risk of hypertension with the adjusted OR (95% CI) of 3.626 (1.609–8.172, *p* = 0.002), but associations of rapid eye movement AHI (REM-AHI) and lowest SaO_2_ with hypertension were not statistically significant.

**Conclusion:**

Increased AHI, NREM-AHI, ODI, and severities of OSAS were significantly associated with higher risks of hypertension in T2DM patients. Detection and treatment of OSAS are needed to prevent hypertension in T2DM patients.

## 1. Introduction

Obstructive sleep apnea syndrome (OSAS), as an increasingly common sleep-related respiratory disease, is characterized by recurrent episodes of upper airway collapse during sleep, bringing about the chronic intermittent hypoxia and sleep fragmentation [1]. And, it is also clinically characterized by snoring, excessive daytime sleepiness, and fatigue. According to population-based studies, OSAS affects 10%–17% of men and 3%–9% of women of the age from 30 to 70 years old [2]. Intermittent hypoxia and hypercapnia during sleep caused by OSAS may result in sympathetic nervous system activation, renin-angiotensin-aldosterone system activation, vascular endothelial dysfunction, oxidative stress, and metabolic dysregulation [3], which are considered to be associated with the development of cardiometabolic diseases such as hypertension and type 2 diabetes mellitus (T2DM) [4, 5].

There is an increasing evidence that OSAS plays a role in the development of T2DM [6], and the prevalence of OSAS is higher in diabetes patients than their controls. An observational study conducted in T2DM patients found that the prevalence rate of OSAS was 86%, of which the moderate and severe OSAS were 30.5% and 22.6%, respectively [7]. Our previous study also found 80.3% of T2DM patients had OSAS which was consistent with others [8]. A community-based study indicated that OSAS was independently related to the development of insulin resistance and could promote the occurrence of T2DM [9]. Both T2DM and OSAS are related to obesity via sharing a common pathophysiological link to the development of insulin resistance [10].

Hypertension is a globally public health problem, and OSAS is one of its modifiable risk factors [11, 12]. Some studies found that there was a significantly positive association between hypertension and OSAS [13, 14]. The prevalence of OSAS is approximately 30–40% in hypertensive patients [15]. Besides, in the resistant hypertensive patients, Sapiña-Beltrán et al [16] found the prevalence rate increased to 83.5%, of which the rates of mild, moderate, and severe OSAS were 31.7%, 25.7%, and 26.1%, respectively. And the adjusted odds ratio of resistant elevated blood pressure (BP) in severe OSAS patients was 4-fold higher [17]. Furthermore, sleep quality scores in patients with hypertension were significantly worse [18]. However, there were significant heterogeneities regarding OSAS assessment in the evidence. Some studies have used polysomnography (PSG), while others were performed by using home sleep apnea testing which has been found to underestimate the severity of OSAS. Therefore, further studies using the more precise assessment of OSAS, such as PSG, should be conducted to clarify the relationship between OSAS and hypertension in T2DM patients.

In the present study, we aimed to explore the independent associations of different PSG indices, such as the apnea-hypopnea index (AHI), rapid eye movement AHI (REM-AHI), nonrapid eye movement AHI (NREM-AHI), the severity of OSAS, and oxygen desaturation index (ODI), and the lowest oxygen saturation (SaO_2_), with risks of hypertension in T2DM patients with adjustment for potential confounding factors.

## 2. Materials and Methods

### 2.1. Study Population

A total of 346 T2DM patients with snoring were recruited from the Department of Endocrinology and Diabetes, the First Affiliated Hospital of Xiamen University (Xiamen, China), between March 2013 and December 2017. Face-to-face interview was conducted to collect some health-related information as before [8]. And we also obtained the written informed consent from each patient. Exclusion criteria included acute illnesses, acute infective diseases, heart diseases, chronic kidney failure, craniofacial abnormalities, and current treatment for breathing disorders. Of them, 316 patients with complete clinical and PSG measurement data were left for further analyses. This study was approved by the Human Research Ethics Committee of the First Affiliated Hospital of Xiamen University (Xiamen, China).

### 2.2. Data Collection

Anthropometric measurements were described in detail previously [19]. Briefly, bodyweight, height, waist circumference (WC), hip circumference (HC), and body mass index (BMI) were measured using a calibrated scale. Waist to hip ratio (WHR) was calculated as WC (cm)/HC (cm). Arterial blood pressure was measured with the OMRON electronic sphygmomanometer, and three readings were taken at 5 min intervals, and the mean of them was recorded. Hypertension was diagnosed as mean systolic BP ≥ 140 mmHg or mean diastolic BP ≥ 90 mmHg [20], or the patients with T2DM had taken antihypertensive drugs before the study.

Fasting blood sample determinations containing fasting plasma glucose (FPG), glycosylated hemoglobin A1c (HbA1c), and lipid profiles (triglyceride (TG), total cholesterol (TC), low-density lipoprotein-cholesterol (LDL-c), and high-density lipoprotein-cholesterol (HDL-c)) were consistent with our previous publication [8]. Electrochemiluminescence immunoassay was used to measure serum fasting insulin concentration (Roche Elecsys Insulin Test, Roche Diagnostics, Mannheim, Germany). Estimated glomerular filtration rate (eGFR) was calculated using the following estimating equation derived from the modification of diet in renal disease (MDRD) equation based on the data from Chinese chronic kidney disease (CKD) patients [21], eGFR (mL/min per 1.73 m^2^) = 175 × Scr (mg/dL)^−1.234^ × age (year)^−0.179^ × 0.79 (if female).

### 2.3. Polysomnography Measurement

Polysomnographic (PSG) measurement (Compumedics, Abbotsford, Australia) was performed by a technician for each patient in the hospital as described before [8]. Evaluation of PSG records was based on the generally accepted scoring methods [22]. The recording time ≥5 h was considered valid; otherwise, it would be repeated the next time. Generally, apnea was defined as reductions in airflow by more than 90% for at least 10 seconds during sleep, and hypopnea was reductions by more than 30% [23]. And the total number of apneas and hypopneas per hour of sleep was termed AHI, which was used for OSAS severity stratification, with values ranging from 5 to 15, 16 to 30, or more than 30 events per hour defined as mild, moderate, or severe, respectively. Some sleep parameters including REM-AHI, NREM-AHI, lowest SaO_2_, and oxygen desaturation index (ODI) were obtained as before [19]. The ODI was defined as the number of times per sleep hour that oxygen saturation dropped by 3% or more [19].

### 2.4. Statistical Analysis

Data were presented as the mean ± standard deviation (SD) or as median (interquartile range) for continuous variables or number (percentage) for categorical variables. All subjects were stratified by the presence of hypertension (normotension or hypertension). Differences between the two groups were analyzed using one-way ANOVA for those with normal distribution or the Kruskal–Wallis test for those with skewed distribution on continuous variables and using the chi-square test on categorical variables.

Multivariable logistic regression was used to calculate adjusted odds ratios (OR) and 95% confidence interval of AHI, REM-AHI, NREM-AHI, and ODI and lowest SaO_2_ for hypertension with adjustment for age, sex, current smoking, regular drinking, BMI, WC, diabetes duration, TC, HDL-c, TG, LDL-c, HbA1c, and eGFR. AHI was presented as continuous variables and OSAS as categorical (mild, moderate, and severe vs. no). ODI was presented as both continuous and categorical variables (tertile 2 and tertile 3 vs. tertile 1). Trend tests for OSAS and ODI as categorical variables were also tested. All *p* values were two sides, and *p* < 0.05 was considered statistically significant. All statistical analyses were performed using SPSS version 21.0 software (IBM Corporation, Armonk, NY).

## 3. Results

### 3.1. Demographic and Clinical Characteristics Categorized by Hypertension

Among the 316 T2DM patients, 220 (69.6%) were men, and the mean ages (±SD) were 52.5 ± 14.0 years. The total prevalence rates of OSAS and hypertension were 64.6% and 41.1%, respectively.  Table 1 shows the differences of demographic and clinical characteristics categorized by hypertension in patients with T2DM. Patients with hypertension were significantly older and had significantly increased levels of T2DM duration and eGFR and the decreased level of HbA1c than their controls. As for the indices of obesity, patients with hypertension also showed significant increases in BMI (28.65 ± 0.37 vs. 27.27 ± 0.28, *p* = 0.003), WC (100.53 ± 0.89 vs. 96.18 ± 0.68, *p* < 0.001), and WHR (0.98 ± 0.01 vs. 0.96 ± 0.01, *p* = 0.003). There was no significant difference in sex, current smoking, regular drinking, lipid profiles (TG, TC, HDL-c and LDL-c), insulin use, and oral glucose-lowering agents (OGLA) between the normotension and hypertension patients.

### 3.2. PSG Indices Categorized by Hypertension

**Figure 1:**
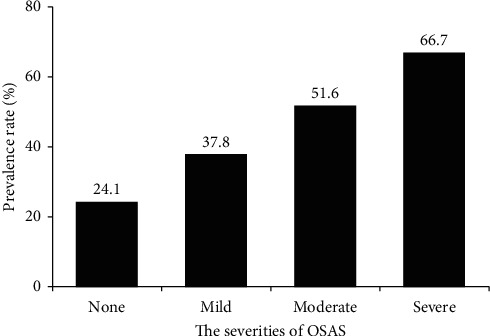
Prevalence of hypertension categorized by severities of OSAS.

Table 2 shows the differences of sleep parameters in normotensive and hypertensive patients with T2DM. The prevalence rates of hypertension increased with the increasing severities of OSAS (no, mild, moderate, and severe, *p* < 0.001) (Figure 1). Subjects with hypertension had significantly lower level of lowest SaO_2_ (74.0 ± 10.4 vs. 77.3 ± 9.8, *p* = 0.004) and higher levels of ODI (48.4 (21.9–78.0) vs. 22.6 (10.8–48.1) events/hour, *p* < 0.001), AHI (17.2 (5.7–34.9) vs. 5.7 (2.1–17.3) events/hour, *p* < 0.001), and NREM-AHI (17.6 (5.5–36.5) vs. 5.2 (2.2–16.6) events/hour, *p* < 0.001) than those with normotension. There was no significant difference in REM-AHI between the two groups (Table 2).

### 3.3. Association of PSG Indices with Hypertension

Increased AHI was significantly associated with higher risk of hypertension, with the unadjusted OR (95% CI) of 1.040 (1.024–1.055, *p* < 0.001). With adjustment for age, sex, current smoking, regular drinking, BMI, WC, diabetes duration, TC, HDL-c, TG, LDL-c, HbA1c, and eGFR, AHI was still significantly associated with the risk of hypertension, and the adjusted OR (95% CI) was 1.026 (1.008–1.044, *p* = 0.004). Similarly, NREM-AHI was also independently associated with the risk of hypertension with the adjusted OR (95% CI) of 1.026 (1.009–1.044, *p* = 0.003). While REM-AHI was not significantly associated with risk of hypertension with the adjusted OR (95% CI) of 1.009 (0.993–1.026, *p* = 0.260) (Table 3).

Compared with non-OSAS subjects, those with mild, moderate, and severe OSAS showed significantly increased risks of hypertension, and the unadjusted ORs (95% CI) were 1.941 (1.027–3.564, *p* = 0.041), 3.358 (1.736–6.497, *p* < 0.001), and 6.296 (3.159–12.584, *p* < 0.001), respectively (Table 3). With adjustment for potential confounders, the significant association between severe OSAS and hypertension was still statistically significant (OR (95% CI): 3.626 (1.609–8.172), *p* = 0.002), although the significant associations of mild and moderate OSAS with hypertension disappeared. There was a significantly positive trend between OSAS severities and risk of hypertension even with adjustment for potential confounding factors (Table 3).

Increased ODI was also significantly associated with higher risk of hypertension, with the adjusted OR (95% CI) of 1.005 (1.001–1.010, *p* = 0.040). As for tertiles of ODI, tertile 3 showed significantly increased risk of hypertension compared with tertile 1, with the adjusted OR (95% CI) of 2.452 (1.222–4.921, *p* = 0.012). Furthermore, there was a significant trend between increased tertiles of ODI and risk of hypertension (trend test: *p* = 0.016), although the significant association of tertile 2, compared to tertile 1, with hypertension disappeared with adjustment for all the potential confounding factors (Table 3).

Higher lowest SaO_2_ was associated with the reduced risk of hypertension with the unadjusted OR (95% CI) of 0.969 (0.947–0.991, *p* = 0.005), while the significant association disappeared after adjustment with the adjusted OR (95% CI) of 0.980 (0.995–1.005, *p* = 0.115) (Table 3).

## 4. Discussion

In the present study, we found that T2DM patients with hypertension showed significantly increased AHI, NREM-AHI, severities of OSAS, and ODI and lowest SaO_2_ than those with normotension. With adjustment for the potential confounding factors, increased AHI, NREM-AHI, and ODI were significantly and independently associated with the increased risk of hypertension, and there were also significantly positive associations of severities of OSAS with risk of hypertension. Associations of REM-AHI and lowest SaO_2_ with hypertension were not statistically significant.

It has been established that OSAS is an important identifiable risk factor of hypertension. The prevalence rate of OSAS in hypertensive subjects of our present study was 79.2%, which was higher than others. A prospective cohort study recruiting 1889 participates showed that OSAS was correlated with the increased risk of hypertension [24], which was consistent with other studies [12, 25]. Guillot et al [26] found that severe OSAS was significantly related to new-onset hypertension after 3 years of follow-up, and the incidence of hypertension increased with the increasing severities of OSAS which was consistent with ours [27]. Continuous positive airway pressure (CPAP) is an effective tool to treat OSAS. Some studies showed that the CPAP treatment for more than 4 hours played a role in lower BP in OSAS patients [28]. However, there were still other studies sharing the opposite perspectives. In a multicenter study with 725 OSAS subjects, CPAP prescription did not decline the incidence of hypertension compared with conventional care [29]. Robinson et al. [30] also reported that in hypertensive OSAS patients, there was no significant decrease in mean 24 h BP after CPAP treatment.

There is emerging evidence that OSAS is more common in patients with T2DM [31]. Insulin resistance, as a key pathogenesis of T2DM, was found to be associated with OSAS. Among 270 nondiabetic patients, fasting serum insulin and homeostasis model assessment (HOMA-IR) were higher in patients with OSAS (AHI ≥5 events/hour) [32]. In addition, AHI ≥5 events/hour was also associated with impaired glucose tolerance and increased risk of diabetes [33]. One study confirmed that OSAS was related to *β*-cells dysfunction [34]. Substantial studies found that the increasing severity of OSAS may also lead to the poorer glucose control [35, 36]. Besides, obesity, as one of the strongest risk factors of OSAS, usually coexisted with OSAS, which would lead to insulin resistance, dyslipidemia, and hypertension [37]. In our study, overweight and obese patients accounted for the majority which would increase the incidence of hypertension. The benefits of CPAP treatment on diabetic and hypertension control are worthy of recognition. A study conducted in T2DM and newly diagnosed OSAS patients showed that CPAP therapy could significantly decrease the sleeping glucose and reach a more stable state [38]. Also, a randomized controlled trail including 59 patients with OSAS and T2DM showed that CPAP therapy was related to the improvement of BP (from 149 ± 23/80 ± 12 to 140 ± 18/73 ± 13) [39].

NREM-related sleep-disordered breathing (NREM-SDB) and REM-related sleep-disordered breathing (REM-SDB) are two different types of sleep-disordered breathing. Approximately, 13.5%–36.7% patients with suspicion of SDB suffered from REM-SDB [40], and some studies indicated that approximately 50% OSAS patients had higher NREM-AHI than REM-AHI [41] which was consistent with ours, and the incidence of severe OSAS was higher in the NREM-SDB group than in the REM-SDB group [42]. There was still controversy in the relationship between REM-AHI with hypertension. Winconsin Sleep Cohort Study [43] revealed that REM-SDB, independent of NREM-SDB, was significantly related to the prevalent hypertension which could be explained by the sympathetic excitement and suppression of genioglossus muscle tone during REM sleep [44, 45]. A study conducted in Korean population with metabolic syndrome found that REM-AHI >15 events/hour could effectively predict the occurrence and development of metabolic syndrome after adjustment for age, BMI, and NREM-AHI ≥15 events/hour [46]. However, some studies showed that there was no significant association between REM-AHI and hypertension in the range of REM-AHI <20 events/hour which was matched with us; the mean values of REM-AHI were 4.3 events/hour in the subgroup of hypertension [47]. Interestingly, both REM-AHI and NREM-AHI were significantly associated with the increased risk of hypertension, but the significant association between REM-AHI and hypertension disappeared after adjustment for potential confounders. These differences may be caused by the following reasons. First of all, the BP values were taken from the average of three measurements, not using the 24 h BP monitoring. Second, we did not stratify the severity of REM-AHI; therefore, the exact relationship of different REM-AHI levels and hypertension was unclear. Third, we did not explore the relationship of REM-AHI and hypertension while limiting the scope of NREM-AHI or adjusting NREM-AHI. Therefore, further research is needed to explore the true association of REM-AHI and hypertension.

There were some limitations in the present study. First, most our subjects with T2DM were overweight or obese, which would increase the risk of hypertension and OSAS (AHI ≥5 events/hour) and may therefore underestimate the true relation between them. Second, the sample size of this study was relatively small and might not have enough power. Third, not all sleep parameters were included in this study. In the end, this research design was cross-sectional and could not draw any causality. Additionally, the subjects with T2DM were not randomly sampled from a database, and there may be selection bias. Therefore, a prospective study with larger sample size should be conducted to explore the true association between OSAS and new-onset hypertension.

## 5. Conclusion

We found that T2DM patients with hypertension showed significantly increased AHI, NREM-AHI, severities of OSAS, and ODI and lowest SaO_2_ than those with normotension. Increased AHI, NREM-AHI, and ODI were significantly and independently associated with the increased risk of hypertension, and there were also significantly positive associations of severities of OSAS with risk of hypertension. Therefore, detection and treatment of OSAS are needed in terms of prevention of hypertension in T2DM patients.

## Figures and Tables

**Table 1:** Demographic and clinical characteristics categorized by hypertension in patients with T2DM.

	All patients	Normotension	Hypertension	*p* value
*n* (%)	316	186 (58.9%)	130 (41.1%)	
Age (years)	52.5 ± 14.0	48.8 ± 1.0	57.9 ± 1.1	<0.001
Sex				
Female (*n*, %)	96 (30.4%)	52 (54.2%)	44 (45.8%)	0.263
Male (*n*, %)	220 (69.6%)	134 (60.9%)	86 (39.1%)	
T2DM duration (years)	4.0 (1.0–10.0)	3 (0.4–8)	6 (2–11)	<0.001
Current smoking (%)	113 (35.8%)	70 (37.6%)	43 (33.1%)	0.406
Regular drinking (%)	100 (31.6%)	55 (29.6%)	45 (34.6%)	0.343
Systolic BP (mmHg)	132.9 ± 17.4	126.92 ± 0.92	141.45 ± 1.74	<0.001
Diastolic BP (mmHg)	80.3 ± 10.2	78.76 ± 0.69	82.60 ± 0.94	0.001
BMI (kg/m^2^)	27.8 ± 4.1	27.27 ± 0.28	28.65 ± 0.37	0.003
Waist circumference (cm)	98.0 ± 9.8	96.18 ± 0.68	100.53 ± 0.89	<0.001
WHR	1.0 ± 0.1	0.96 ± 0.01	0.98 ± 0.01	0.003
HbA1c (%)	9.9 ± 2.2	10.16 ± 0.16	9.58 ± 0.20	0.023
Triglyceride (mmol/L)	1.7 (1.2–2.5)	1.62 (1.14–2.49)	1.78 (1.33–2.52)	0.125
Total cholesterol (mmol/L)	5.2 ± 1.1	5.19 ± 0.08	5.16 ± 0.11	0.826
HDL-cholesterol (mmol/L)	1.0 (0.9–1.2)	1.02 (0.91–1.20)	1.03 (0.90–1.18)	0.465
LDL-cholesterol (mmol/L)	3.2 ± 1.0	3.18 ± 0.08	3.12 ± 0.09	0.585
eGFR	110.8 ± 31.9	115.75 ± 2.23	103.77 ± 2.96	0.001
Insulin use (%)	206 (65.2%)	115 (64.2%)	91 (71.7%)	0.173
OGLA use (%)	249 (78.8%)	145 (78.0%)	104 (80.0%)	0.662
Statin use (%)	234 (74.1%)	125 (67.6%)	109 (83.8%)	0.001
Anticoagulation use (%)	178 (56.3%)	84 (45.2%)	94 (72.3%)	<0.001

Data were presented as the mean ± SE or as median (interquartile range) for continuous variable or number and percentage for categorical variable. AHI, apnea-hypopnea index; BMI, body mass index; BP, blood pressure; HDL, high-density lipoprotein; LDL, low-density lipoprotein; NREM, nonrapid eye movement; ODI, oxygen desaturation index; OGLA, oral glucose-lowering agents; OSAS, obstructive sleep apnea syndrome; PSG, polysomnography; REM, rapid eye movement; SaO_2_, arterial oxygen saturation; T2DM, type 2 diabetes mellitus.

**Table 2:** PSG indices categorized by hypertension in patients with T2DM.

	All patients	Normotension	Hypertension	*p* value
*n* (%)		186 (58.9%)	130 (41.1%)	
AHI	9.0 (3.3–24.9)	5.7 (2.1–17.3)	17.2 (5.7–34.9)	<0.001
REM-AHI (events/h)	2.4 (0.0–15.8)	1.9 (0.0–12.7)	4.3 (0.0–20.4)	0.142
NREM-AHI (events/h)	8.8 (3.3–25.9)	5.2 (2.2–16.6)	17.6 (5.5–36.5)	<0.001
OSAS categories				<0.001
No	112	85 (75.9%)	27 (24.1%)	
Mild	82	51 (62.2%)	31 (37.8%)	
Moderate	62	30 (48.4%)	32 (51.6%)	
Severe	60	20 (33.3%)	40 (66.7%)	
ODI (events/h)	32.5 (13.9–62.9)	22.6 (10.8–48.1)	48.4 (21.9–78.0)	<0.001
Lowest SaO_2_ (%)	76.0 ± 10.2	77.3 ± 9.8	74.0 ± 10.4	0.004

AHI, apnea-hypopnea index; CI, confidence interval; NREM, nonrapid eye movement; ODI, oxygen desaturation index; OR, odds ratio; OSAS, obstructive sleep apnea syndrome; REM, rapid eye movement; SaO_2_, arterial oxygen saturation; T2DM, type 2 diabetes mellitus.

**Table 3:** Unadjusted and adjusted odds ratios (ORs) with associated 95% confidence interval (CI) for hypertension in patients with T2DM.

Variables	Unadjusted OR			Adjusted OR^∗^		
	OR	95% CI	*p* value	OR	95% CI	*p* value
AHI (events/h)	1.040	1.024–1.055	<0.001	1.026	1.008–1.044	0.004
REM-AHI (events/h)	1.019	1.004–1.034	0.012	1.009	0.993–1.026	0.260
NREM-AHI (events/h)	1.040	1.025–1.055	<0.001	1.026	1.009–1.044	0.003
OSAS categories						
None	1.000			1.000		
Mild	1.914	1.027–3.564	0.041	1.360	0.669–2.765	0.396
Moderate	3.358	1.736–6.497	<0.001	1.656	0.765–3.585	0.200
Severe	6.296	3.159–12.548	<0.001	3.626	1.609–8.172	0.002
Trend test			<0.001			0.002
ODI						
ODI (events/h)	1.010	1.004–1.016	0.001	1.005	1.000–1.010	0.040
Tertiles of ODI						
Tertile 2 vs. tertile 1	2.674	1.464–4.885	0.001	1.730	0.867–3.452	0.120
Tertile 3 vs. tertile 1	5.024	2.750–9.178	<0.001	2.452	1.222–4.921	0.012
Trend test			<0.001			0.016
Lowest SaO_2_ (%)	0.969	0.947–0.991	0.005	0.980	0.995–1.005	0.115

^∗^OR was adjusted for age, sex, current smoking, regular drinking, BMI, WC, diabetes duration, total cholesterol, HDL-cholesterol, triglyceride, LDL-cholesterol, HbA1c, and eGFR. AHI, apnea-hypopnea index; CI, confidence interval; NREM, nonrapid eye movement; ODI, oxygen desaturation index; OR, odds ratio; OSAS, obstructive sleep apnea syndrome; REM, rapid eye movement; SaO_2_, arterial oxygen saturation; T2DM, type 2 diabetes mellitus.

## Data Availability

The datasets used and/or analyzed during the current study are not publicly available due to some privacy reasons and may be available from the corresponding author upon reasonable request.
